# Supplementary material for: MetaRibo-Seq measures translation in microbiomes
Source: Nat Commun. 2020 Jun 29;11:3268. doi: 10.1038/s41467-020-17081-z (PMC7324362; doi:10.1038/s41467-020-17081-z)
Supplement: Supplementary file 10 — Supplementary Data 7 [file 41467_2020_17081_MOESM10_ESM.zip › File2/Confidence_High_Taxonomy/57067_out.krona.html]

Javascript must be enabled to view this page.

members
magnitude
magnitudeUnassigned
count
unassigned
taxon
rank

57067\_out

24

2
superkingdom
24

1239
phylum
24

186801
class
24

24
186802
order

24

SRS011084\_contig\_number\_28933SRS014613\_contig\_number\_3235SRS016018\_contig\_number\_4315SRS019381\_contig\_number\_22087SRS020869\_contig\_number\_35288SRS049959\_contig\_number\_48122SRS050752\_contig\_number\_27243SRS054905\_contig\_number\_6711SRS056259\_contig\_number\_15694SRS064276\_contig\_number\_contig-100\_29310.124988SRS075984\_contig\_number\_contig-100\_7211.87985SRS077641\_contig\_number\_contig-100\_11391.88274SRS098061\_contig\_number\_3782SRS098571\_contig\_number\_contig-100\_6179.218164SRS104400\_contig\_number\_18617SRS104912\_contig\_number\_4458SRS105153\_contig\_number\_34509SRS142542\_contig\_number\_contig-100\_3584.132186SRS143523\_contig\_number\_contig-100\_24166.72247SRS143991\_contig\_number\_19603SRS144537\_contig\_number\_50032SRS147346\_contig\_number\_39819SRS147425\_contig\_number\_contig-100\_1955.1956SRS147614\_contig\_number\_6200
1898207
species
